# Complete Genome Sequence of Escherichia coli Siphophage Snoke

**DOI:** 10.1128/MRA.01051-19

**Published:** 2019-10-03

**Authors:** James E. Corban, Jacob Gramer, Russell Moreland, Mei Liu, Jolene Ramsey

**Affiliations:** a Center for Phage Technology, Texas A&M University, College Station, Texas, USA; Loyola University Chicago

## Abstract

Escherichia coli is a Gram-negative bacterium often found in animal intestinal tracts. Here, we present the genome of the *Guernseyvirinae*-like E. coli 4s siphophage Snoke. The 44.4-kb genome contains 81 protein-coding genes, for which 33 functions were predicted. The capsid morphogenesis gene in Snoke contains a large intein.

## ANNOUNCEMENT

Escherichia coli, a heavily studied Gram-negative bacterium, is most commonly found in the intestinal tract of humans and other animals (1). Selective pressures generated from bacteriophage-bacterium interactions in the gut shape the development of both organisms (2). Studying bacteriophages that grow on E. coli 4s (3), a horse fecal isolate, may illuminate the mechanisms of phage-bacterium coevolution within unique natural microbiomes (4). Here, we present the complete genome sequence of the E. coli 4s siphophage Snoke.

Bacteriophage Snoke was isolated from filtered (0.2-μm-pore-size) activated sludge sourced from a wastewater treatment facility in Austin, TX. The phage was propagated on E. coli 4s aerobically at 37°C in Luria broth (BD) using the soft-agar overlay methods described in reference 5. Genomic DNA was purified from the phage as previously described with the Promega Wizard DNA clean-up system (6), prepared as Illumina TruSeq Nano low-throughput libraries, and sequenced in paired-end 250-bp reads using v2 500-cycle chemistry on an Illumina MiSeq platform. The 565,076 sequence reads from the index containing the phage genome were quality controlled with FastQC (http://www.bioinformatics.babraham.ac.uk/projects/fastqc/), and the phage genome was assembled into a single raw contig via SPAdes v.3.5.0 with 1,291.6-fold coverage after trimming using the FASTX-Toolkit 0.0.14 (http://hannonlab.cshl.edu/fastx_toolkit/) (7). PCR amplification across the raw contig ends (forward primer 5′-GCAACACGGCACAGAAAC-3′ and reverse primer 5′-CTGCGACGGAGAAATCAACT-3′) and verification by Sanger sequencing of the DNA product ensured that the contig sequence was complete. Accompanied with manual corrections, protein-coding gene predictions were performed using GLIMMER v3.0 and MetaGeneAnnotator v1.0; predicted gene functions were assigned using InterProScan v5.33-72, the HHSuite v3.0 HHpred tool (multiple sequence alignment [MSA] generation with the HHblits ummiclus30_2018_08 database and modeling with PDB_mmCIF70), BLAST v2.2.31 with a 0.001 maximum expectation value, and TMHMM v2.0 at the default settings (8–13). All BLAST queries were run against the NCBI nonredundant and UniProtKB Swiss-Prot and TrEMBL databases (14). ARAGORN v2.36 was run to detect tRNA coding sequences (15). Rho-independent termination sites were annotated using TransTermHP v2.09 (16). DNA sequence similarity was determined using the progressiveMauve v2.4.0 alignment algorithm (17). The genome annotation tools (with the exception of HHpred) were accessed via the Center for Phage Technology Galaxy and Web Apollo interfaces (https://cpt.tamu.edu/galaxy-pub) and run with default parameters, unless stated above (18, 19). The morphology of bacteriophage Snoke was determined using samples negatively stained with 2% (wt/vol) uranyl acetate and viewed by transmission electron microscopy at the Texas A&M Microscopy and Imaging Center (20).

The 44,454-bp genome of Snoke has a G+C content of 50.2% and 95.0% coding density. Genome analysis indicates 81 protein-coding genes, of which 33 received a functional annotation, but no tRNA genes. The Snoke genome is predicted by PhageTerm to be packaged via a pac-type (headful) DNA packaging mechanism (21). Snoke shares 69.0% nucleotide sequence identity and 58 proteins with *Escherichia* phage VB_EcoS-Golestan (GenBank accession no. MG099933), a member of the *Guernseyvirinae* subfamily (22).

A large self-splicing intein resides within the capsid morphogenesis protein (NCBI accession no. QEG06950) (23). The capsid morphogenesis protein in Salmonella phage LSPA1 (GenBank accession no. KM272358) contains a nearly identical intein, and K1ind2 (GenBank accession no. GU196280) lacks the intein.

### Data availability.

The genome sequence and associated data for phage Snoke were deposited under GenBank accession no. MK931441, BioProject accession no. PRJNA222858, SRA accession no. SRR8892143, and BioSample accession no. SAMN11408679.
